# Flow-induced Klf4-Akt signaling links EC cycling to mural cell defects in arterial-venous malformations

**DOI:** 10.7150/thno.121154

**Published:** 2026-02-26

**Authors:** Yanzhu Lin, Zohrah Hashemi, Qing Zhang, Yuxi Di, Tanmaya Behera, Johannes Gahn, Kuheli Banerjee, Fan Wu, Kornelia Andorfer, Mahak Singhal, Caroline Seebauer, Roxana Ola

**Affiliations:** 1 Experimental Pharmacology Mannheim (EPM), European Center for Angioscience (ECAS), Medical Faculty Mannheim, Heidelberg University, Germany.; 2 Department of Nuclear Medicine, First Affiliated Hospital of Sun Yat-Sen University, Guangzhou, China.; 3 Department of Otorhinolaryngology, Regensburg University Medical Center, Regensburg, Germany.; 4 European Center for Angioscience, Medical Faculty Mannheim, Heidelberg University, Mannheim, Germany; Helmholtz-Institute for Translational AngioCardioScience (HI-TAC) of the Max Delbrück Center for Molecular Medicine in the Helmholtz Association (MDC), Heidelberg University, Heidelberg, Germany.; 5 Department of Otorhinolaryngology, Head and Neck Surgery, Luzerner Kantonsspital, Lucerne, Switzerland.

**Keywords:** shear stress, mural cells, cell cycle, AVM, HHT, BMP signaling.

## Abstract

Fluid shear stress (FSS) safeguards vascular homeostasis, coordinating endothelial cell (EC) behavior and endothelial - mural cell communication. Disrupted flow sensing driving excessive proliferation contribute to arterial-venous malformations (AVMs) in Hereditary Hemorrhagic Telangiectasia (HHT) vascular disorder. Yet, how flow-dependent cell cycle regulation intersects with mural cell remodeling in HHT remains unclear.

**Methods:**

We used a combination between *in vitro* shear stress assays and *in vivo* analyses of multiple murine HHT models, including endothelial-specific loss of Activin-like kinase 1 (*Alk1)* or *Smad4* and bone morphogenic factor 9/10 (BMP9/10) ligand blockade. Retinal vasculature and human nasal mucosal biopsies from HHT2 patients were examined for pathway conservation. Endothelial - mural cell crosstalk was evaluated using transwell and three-dimensional flow-dependent co-culture assays. Loss and gain of function studies were employed to define disease mechanisms.

**Results:**

Across all studied murine HHT models and in HHT2 telangiectasias, AVM endothelium exhibited excessive flow-induced Krüpper-like 4 (KLF4) - Akt pathway activation, sustained EC proliferation, and abolition of FSS-mediated cyclin-dependent kinases 2/6 (CDK2/6) inhibition. The hyperproliferative state suppressed the expression of endothelial platelet-derived growth factor B (PDGFB) leading to pericyte loss, and and mural cell remodeling in AVMs. Restoration of endothelial quiescence via inhibition of KLF4, Akt or CDK4/6 rescued FSS-induced PDGFB expression. Pharmacological PDGFB induction with thalidomide restored mural cell coverage, and significantly reduced AVM burden *in vivo*.

**Conclusion:**

Our study establishes EC cycle state as the upstream determinant of mural cell stability under pathological flow and provides the mechanistic reasoning for why distinct therapeutic strategies (e.g., CDK4/6 inhibition, Akt modulation, or thalidomide-induced PDGFB upregulation) converge on AVM stabilization.

## Introduction

Fluid shear stress (FSS), a mechanical force generated by blood flow guides vascular development, remodeling and homeostasis 1. Throughout life, endothelial cells (ECs) adapt and respond to small and short-term changes in FSS by altering their morphology, number, fate, and orientation, thereby modulating vessel caliber and structure. These adaptive responses occur through autocrine mechanisms involving EC mechanotransduction pathways 2, and paracrine signaling to perivascular mural cells, including pericytes (PCs) and smooth muscle cells (SMCs) 3. In ECs, FSS modulates expression of mural cell recruitment factors, including platelet-derived growth factor B (PDGFB) 4. Endothelial PDGFB acting though PDGF receptor β (PDGFRβ) on PCs plays a critical role in pericyte recruitment and maintenance on developing vessels 5, 6. Once enwrapping capillaries, PCs provide structural and functional support. Contractile PCs constrain capillary EC size and diameter, regulating blood pressure 7, 8 and flow directionality that dictates ECs migratory cues 5 and fate 9, while fine-tuning nutrient and gas exchange within surrounding tissues 10. Whether these angiocrine-paracrine mediated cell events are inter-connected, and whether distinct mechanotransduction pathways coordinately regulate these processes, remains largely unknown.

FSS-mediated cell cycle arrest is essential for the specification of the arterial EC fate 11. Disruption of these events through aberrant activation of the Krüpper-like 4 (Klf4)-Akt activation leads to arterial-venous malformations (AVMs) in mice with EC specific *Smad4* loss of function (LOF) 12. AVMs are dilated, fragile and leaky direct connections between arteries and veins and a pathological hallmark of Hereditary Hemorrhagic Telangiectasia (HHT), an inherited vascular syndrome attributable to LOF heterozygous mutations in genes encoding the bone morphogenic factors (BMP) 9/10 receptors: Activin like kinase 1 (*ALK1,* HHT2) and Endoglin (*ENG*; causing HHT1) and the transcriptional factor* SMAD4,* which underlies the juvenile polyposis (JP)-HHT overlap syndrome 13-15.

Although early studies attributed AVM formation to increased EC size and impaired flow-migration coupling 16-18, recent evidence indicates that dysregulated EC proliferation is the primary initiating event common to all HHT models 12, 19-21.

Murine HHT-like AVMs also exhibit impaired mural cell coverage 4, 22-28, and enforced PDGFB expression has been shown to enhance pericyte recruitment, stabilize vessels integrity and reduce hemorrhage in both experimental models 29-31, and HHT patients 30, 32, 33.

Here, we investigated whether FSS-mediated cell cycle arrest and mural cell remodeling are mechanistically interconnected. We identified a heightened Klf4-Akt-cyclin-dependent kinases 2/6 (Cdk2/6) signaling cascade leading to excessive proliferation in murine AVMs across multiple HHT models and in mucosal AVMs from HHT2 patients. This dysregulation suppresses FSS-induced PDGFB expression, leading to pericyte dropout, mural cell remodeling, and the formation of structurally unstable AVMs. These findings uncover a mechanistic link between flow-mediated cell cycle regulation and mural cell homeostasis via the Klf4-Akt-Cdk-PDGFB signaling axis. Therapeutic strategies that restores flow-dependent endothelial quiescence or reestablish PDGFB-mediated pericyte communication via agents such as thalidomide and/or Cdk inhibitors hold promise for preventing or stabilizing AVMs in HHT patients.

## Materials and Methods

### Mice

Eight weeks old *Alk1^fl/fl^*, *Smad4^ fl/fl^* and Cadherin 5 (*Cdh5)-Cre^ERT2^* mice in a mixed genetic background were intercrossed for experiments to obtain tamoxifen (Tx) inducible EC specific *Alk1* and *Smad4* knockout (*Alk1^iΔEC^* and* Smad4^iΔEC^*) neonates. Intra-peritoneal (i.p.) injections with 100 μg Tx (2 mg/ml, T5648, Sigma-Aldrich) dissolved in corn oil in 50 µl final volume in pups at postnatal day 4 (P4) in *Alk1^iΔEC^* and at P1-P3 in *Smad4^iΔEC^* was used to induce gene deletion. Tx-injected *Cre* negative littermates were used as experimental controls. BMP9/10 blocking antibodies (BMP9/10 blABs) (gift from Genentech, 10 mg kg^-1^ per day) were injected i.p. at P2-P4. All mice were analyzed at P6. 70 mg/kg/day Thalidomide (Sigma, T144) was injected i.p. at P4, P5 and retinas were analyzed at P6. Animal procedures were approved by the Regierungspräsidium Karlsruhe animal welfare commission. Mice were maintained in a pure C57BL/6J background and both sexes were used indistinctively.

### Cell cultures

Human umbilical vein endothelial cells (HUVECs) were isolated from umbilical cords of healthy, consented pregnant women and cultured in EC medium (EGM-2, C-22022; Promocell) supplemented with 5% fetal bovine serum (FBS) (F-7524, Sigma-Aldrich).

Gene depletion was achieved by transfecting 25 pmol siRNAs with Lipofectamine RNAiMax (Invitrogen), following the manufacturer's instructions. Transfection efficiency was assessed by western blotting (WB) and quantitative Polymerase Chain Reaction (qPCR). Experiments were performed 48 -72 hours (h) post-transfection and results were compared with siRNA control (*CTRL* siRNA (D-001810-01-05)). The following siRNAs were used for experimentation: ON-TARGET plus *SMAD4* siRNA (L-003902-00-0005, Dharmacon), ON-TARGET plus *ALK1* siRNA (L-005302-02, Dharmacon), ON-TARGET plus *ENG* siRNA (SI02663024, Qiagen), ON-TARGET plus *CDK2* siRNA (L-003236-00-0005, Dharmacon), ON-TARGET plus *CDK6* siRNA (L-003240-00-0005, Dharmacon) and ON-TARGET plus *PDGFB* siRNA (L-011749-00-0005, Dharmacon). BMP9/10 BlAbs were used at a concentration of 1 ng/ml each, and added every 24 h in complete medium.* CTRL, KLF4* and *AKT* overexpression (OE) cell lines were obtained as previously described 12.

Phosphatidylinositol-4,5-Biphosphate 3-Kinase (PI3K) inhibitor: Pictilisib (Selleckchem, 20 nM), CDK4/6 inhibitor (Palbociclib, 2 μM), Thalidomide (Selleckchem, 100 μM), all diluted in DMSO, were added to cells subject to 12 dynes/cm² FSS for 24 h. Corresponding volumes of DMSO were added to *CTRL* cells.

### Exposure to shear stress

siRNAs or OE-HUVECs plated in 6-well plates were placed on an orbital shaker (Rotamax120, Heidolph Instruments) at a rotation per minute (rpm) rate of 220 in order to generate laminar shear stress of 12 Dynes/cm^2^. qPCR results were confirmed using a pump system (μ-Slide VI^0.4^ - Ibidi).

### Quantitative real-time PCR

RNA was extracted with RNeasy Mini Kit (Qiagen) according to the manufacturer's protocol. Upon measurement of the RNA concentration using a NanoDrop ND-1000 spectrophotometer, 1μg RNA was reverse transcribed according to the manufacturer's protocol (4368813, Thermo Fisher). The differences in mRNA expression were determined by ΔCT against an internal control. RNA was purified using RNeasy-kit (Qiagen) and transcribed using the High-Capacity cDNA Reverse Transcription Kit (Thermo Fisher Scientific). Using PowerUP SYBR Green Master Mix (Thermo Fisher Scientific), qPCR was performed according to the manufacture's protocol. Human primer sequences used: *KLF4*: fw: CCCACATGAAGCGACTTCCC and rev: CAGGTCCAGGAGATCGTTGAA; *KLF2* (Qiagen); *PDGFB*: fw: CTCGATCCGCTCCTTTGATGA and rev: CGTTGGTGCGGTCTATGAG, *CDK2*: fw: CCAGGAGTTACTTCTATGCCTGA and rev: TTCATCCAGGGGAGGTACAAC; *CDK6:* fw: TCTTCATTCACACCGAGTAGTGC and rev: TGAGGTTAGAGCCATCTGGAAA; *JAG1*: fw: GTCCATGCAGAACGTGAACG and rev: GCGGGACTGATACTCCTTGA; *TGFb1*: fw: CAATTCCTGGCGATACCTCAG and rev: GCACAACTCCGGTGACATCAA.

**Retinas:** Eyes were removed from P6 pups and fixed in 4% paraformaldehyde (PFA) for 17 minutes (min) at room temperature (rt). Staining with specific antibodies (**Table S1**) was performed as previously described 12.

### Proliferation assay

Proliferation analysis was performed as described 12.

### Human samples

Nasal biopsies containing AVMs surrounded of normal tissue from patients, who underwent surgery for vascular malformations associated with HHT between 2018 - 2023 was conducted in the Department of Otorhinolaryngology, University Medical Center Regensburg, Germany, in accordance with the principles of the Declaration of Helsinki and was approved by the local ethics committee (No. 17-854-101). Written informed consent was obtained from all participants. Tissues were subsequently embedded in paraffin. Serial sections (5 μm) were deparaffinized in xylene and rehydrated via decreasing ethanol concentrations (Carl Roth). Hematoxylin (nuclei)/Eosin (cytoplasm) (H&E, Sigma Aldrich) and immunofluorescence staining was performed in subsequent sections. Following deparaffination and rehydration, the slides were placed in working solution at PH 6.0 for antigen retrieval and treated with 3% H_2_O_2_. Blocking was performed using 5% BSA for 15 min. The primary antibodies were then incubated overnight at 4 °C and washed with PBST for three times. Slides were then incubated with secondary antibody for 1.5 h at rt in the dark. The used antibodies are included in **Table S2**. Slides were afterwards mounted with mounting medium and imaged with the LSM800 Confocal microscope with Airyscan Detector and the Zeiss ZEN software. Quantification of retina and human samples was done using Fiji.

### Western blotting

Total proteins were lysed with Laemmli buffer (1610747, Bio-Rad) and equal amounts were separated using polyacrylamide separation and electrophoresis and transferred (semi-dry) to a nitrocellulose membrane. Membranes were washed and incubated with the primary antibodies overnight at 4 °C. The following day, the blots were washed and incubated with the secondary antibodies, HRP-conjugated for 1h at rt. Western blots were developed with the Clarity Western ECL Substrate (Bio-Rad) on a Luminescent image Analyzer, Fusion FX (Vilber). Bands intensity was quantified using Image J. The antibodies used are included in **Table S3.**

### Pericyte migration assays

In transwell migration assays, *CTRL* or *SMAD4* siRNAs HUVECs, or HUVECs transduced with* CTRL* OE, *KLF4* OE, or *AKT* OE lentiviruses, were seeded in 24-well transwell plates (8-μm pore size; Corning) and cultured for 24 h prior to the assay. Primary human placental PCs (PromoCell, 2 × 10⁴ cells per insert) were seeded in the upper chambers. Recombinant human PDGFB (100 ng/ml; R&D) versus PBS was added to evaluate PDGFB-dependent pericyte migration. After 24 h of incubation at 37 °C, non-migrated PCs on the upper surface of the membrane were gently removed using cotton swabs. Migrated PCs on the lower surface were fixed with 4% PFA, stained with phalloidin and DAPI, and imaged using an inverted confocal microscope (ZEISS LSM 880). For pericyte migration assay performed under FSS, 2 × 10⁵ cells/gel primary human placental PCs were suspended in 1.5 mL fibrinogen solution (2.5 mg/mL; Sigma-Aldrich) prepared in EBM-2 supplemented with 1% FBS and 50 µg/mL aprotinin (Sigma-Aldrich). The fibrin mixtures were casted into 6-well plates, and gel polymerization was initiated by adding 1 U thrombin (Sigma-Aldrich). After 30 min of incubation at 37 °C, HUVECs transfected with* CTRL*, *SMAD4* or *PDGFB* siRNAs were seeded on top of the fibrin gels and allowed to adhere for 60 min. Wells were then filled with complete EGM medium and cultured overnight. The following day, endothelial-pericyte co-cultures were exposed to laminar shear stress (12 dynes/cm²) for 48 h in medium containing 10% FBS. Cells were subsequently fixed in 3.7% PFA for 2 h, permeabilized with 1% Triton X-100 for 15 min, and stained overnight with DAPI, phalloidin and alpha smooth muscle actin (α-SMA). Fibrin gels were carefully mounted on glass slides using Fluoromount-G and covered with coverslips. All experiments were performed in triplicate. Imaging was performed using a ZEISS LSM 880 inverted confocal microscope using a 10x objective. Z-stacks (200-µm total thickness, 1-µm step size) were acquired starting from the endothelial layer. Pericyte migration was quantified from YZ projections of 200-µm stacks as the distance (µm) between pericytes within the fibrin gel and the overlying HUVEC monolayer. Quantification of % of PCs with α-SMA fluorescence was calculated as a ratio of the threshold value of α-SMA^+^ immunofluorescence per threshold value of phalloidin^+^ PCs in XY projections of the 200-µm stacks.

### Image analysis

Inverted Confocal ZEISS LSM 880 was used for acquiring all images (not-blinded) and Fiji was used for image analysis and quantifications. For quantifying the number of Klf4^+^ ECs in vascular plexus capillaries, the ratio of the number of Klf4^+^ and Early growth response factor 1 (Erg1)^+^ ECs per total number of Erg1^+^ ECs was calculated. The vascular plexus capillary EC area was quantified as threshold value of IB4^+^ endothelial area per total field area. Quantification of phosphorylated S6 ribosomal protein (pS6) in retina vascular plexus capillaries was calculated by normalizing pS6 fluorescence intensity threshold value per IB4^+^ vascular plexus capillary area. For EC proliferation: the ratio of total number of EdU^+^ Erg1^+^ ECs per total number of Erg1^+^ cells in the vascular plexus capillaries was calculated; or the total number of KI67^+^ ECs per vascular plexus capillaries area. Pericyte area in the vascular plexus capillaries was quantified as a threshold value of pericyte marker neural/glial antigen 2 (NG2)^+^ or PDGFRß^+^ immunofluorescence. To quantify pericyte coverage in the vascular plexus capillaries, the pericyte area (%) per total capillary EC area was calculated. Vascular SMC area in retina vasculature was quantified as a threshold value of α-SMA^+^ immunofluorescence. Quantification of vascular SMC coverage in the retina vascular plexus capillaries was calculated by normalizing α-SMA^+^ area to EC capillary area. In the pericyte chemotaxis experiments, pericyte area was quantified as a threshold value of phalloidin immunofluorescence per total field area. Using PECAM membrane staining, 30 capillary EC sizes were measured per capillary field, and the average size was plotted. To determine colocalization between NG2^+^ and PDGFRß^+^ immunostaining, we used the ImageJ plugin “JACop” and calculated the Mander's colocalization coefficient M2 that represents the percentage of PDGFRß^+^ that is present in NG2^+^ PCs. Quantifications were performed in 3 retinas per group.

In human samples, the arteries were distinguished from the veins by using vascular SMC layer's width as a reference value. For quantifying the number of KLF4^+^ ECs in human sections, the ratio of KLF4^+^ ERG1^+^ ECs per total number of ERG1^+^ cells, or ratio of number of KLF4^+^ ECs per total number of DAPI^+^ ENG^+^ in AVM regions, arterial and venous ECs, respectively, was calculated. Quantification of proliferating ECs in human sections was calculated as the ratio of the number of PCNA^+^ ECs per number of DAPI^+^ ENG^+^ ECs. Quantification of pS6 was calculated as a threshold value of pS6 fluorescence intensity per ENG^+^ vascular area. Vascular SMC layer thickness was calculated by measuring the diameter of α-SMA^+^ layer (μm) along the vessel length in AVM regions, arterial and venous ECs, respectively.

### Secondary antibodies

For WB: all antibodies were purchased from Vector Laboratories and used in a concentration of 1:10.000: Anti-Goat Peroxidase (PI-9500), Anti-Rabbit (PI-1000), Peroxidase Anti-Mouse Peroxidase (PI-2000). For IF: Alexa Fluor Anti Goat (A11055/A11057/A21447), Alexa Fluor Anti Rabbit (A10042/A21206), Alexa Fluor Anti Mouse (A31571/A21202) and Hoechst 33342 (1:1000, C10337G), all from Invitrogen in dilution of 1:500.

### Statistical analysis

Data are expressed as means ± SEM. Statistical significance for paired samples and for multiple comparisons was determined by Mann-Whitney and ANOVA, respectively. 2-way Anova (Tukey's multiple comparison test was used for all two factors comparisons), with the adjusted p-values in figure legends and F statistic and the corresponding p-values in the Data S1.xl File. Data were considered to be statistically significant if the adjusted p-value was < 0.05.

## Results

### Canonical BMP9/10 signaling pathway restrains flow-induced KLF4-Akt activation *in vitro*

**Figure 1:**
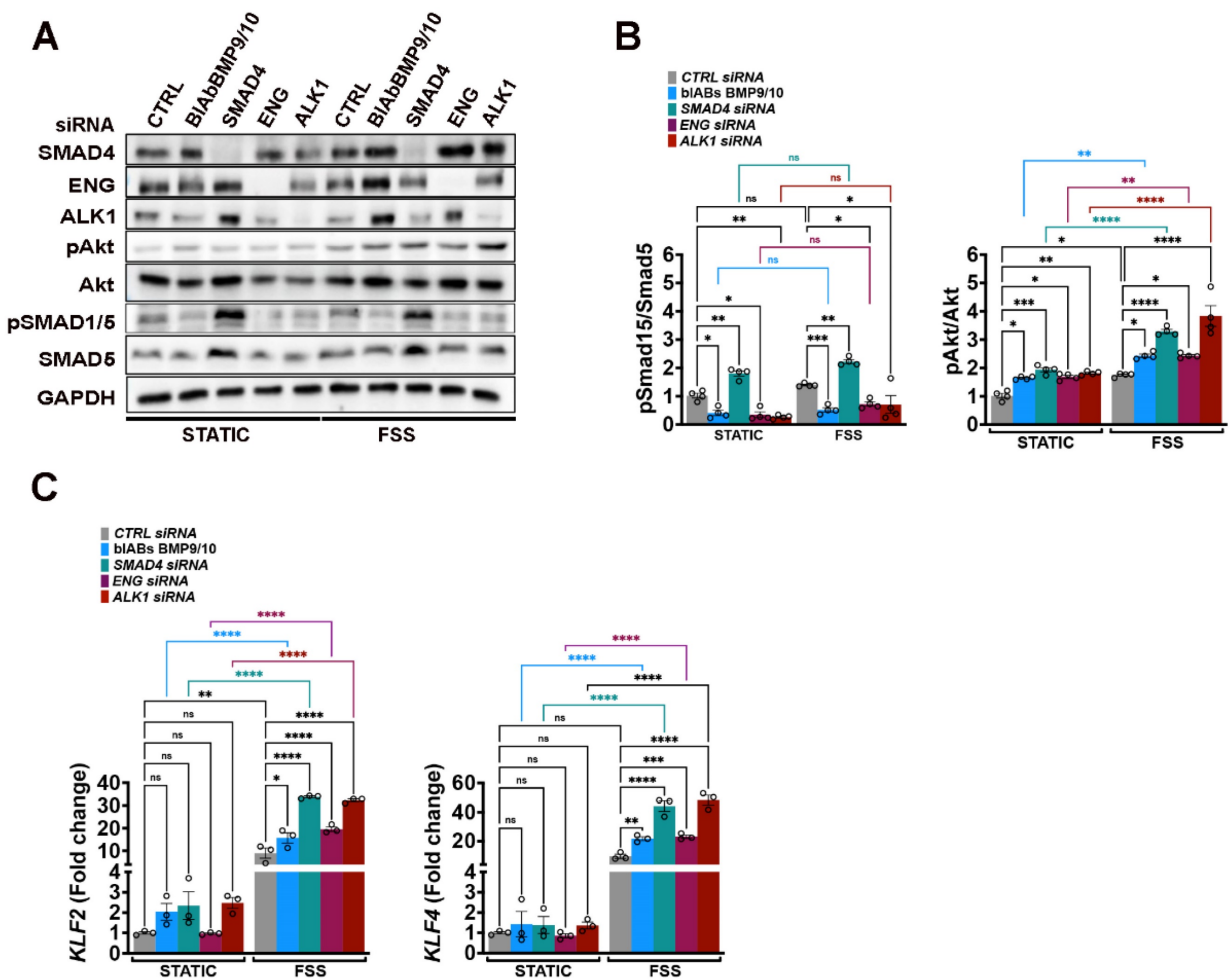
** Canonical BMP9/10 signalling restricts FSS induced KLF4-pAkt *in vitro.* (A)** WB for the indicated proteins in *CTRL*, *SMAD4, ENG* and *ALK1* siRNAs HUVECs or HUVECs treated with BMP9/BMP10 BlAb grown in complete medium conditions in static or subject to 12 Dynes/cm^2^ FSS for 2 h. **(B)** Quantifications of pAkt and pSMAD1/5 levels normalized to total Akt and SMAD5, respectively, for the indicated conditions (n = 4 independent experiments/group).** (C)** *KLF2* and *KLF4* fold change in *CTRL*, *SMAD4, ENG* and *ALK1* siRNAs HUVECs or HUVECs treated with BMP9/BMP10 BlAb grown in static or subject to 12 Dynes/cm^2^ FSS for 2 h (n = 3 independent experiments/group). 2-way Anova with Tukey's multiple comparison test in **B**, **C** was used to determine statistical significance. Data are represented as mean ± SEM with the adjusted p-values ns: non-significant, *P < 0.05, **P < 0.01, ***P < 0.001, ****P < 0.0001.

In the HHT-JP murine model, enhanced Akt activation resulting from disrupted Phosphate and Tensin Homolog (PTEN) - mediated PI3K hydrolysis, further amplified by aberrant Klf4 induction under pathological FSS drives AVM formation 12. To determine whether this mechanism is restricted to endothelial *Smad4* deficiency or it reflects a broader consequence of impaired canonical BMP9/10 signaling, we inactivated the pathway in HUVECs using siRNAs targeting *ENG*, *ALK1*, or *SMAD4*, and by applying BlAb BMP9/10 alongside *CTRL* siRNA. Cells were grown in static conditions or subject to physiological FSS (12 Dynes/cm²) for 2 h (**Figure 1A-C**). Efficient disruption of BMP9/10 signaling was confirmed by WB for phosphorylated SMAD1/5 (pSMAD1/5). Inactivation of BMP9/10 canonical pathway at any level moderately increased the basal Akt activation, which was further exacerbated under FSS (**Figure 1A**-**B**) due to augmented flow-induced Krüppel-Like Factor 2 (*KLF2*) and *KLF4* expression (**Figure 1C**). Collectively, these findings indicate that both, basal and FSS stimulated Akt activation are restrained by the canonical BMP9/10-Alk1/Eng-Smad4 signaling pathway.

### Overactivated Klf4-Akt activation characterizes retinal HHT-like AVMs

**Figure 2:**
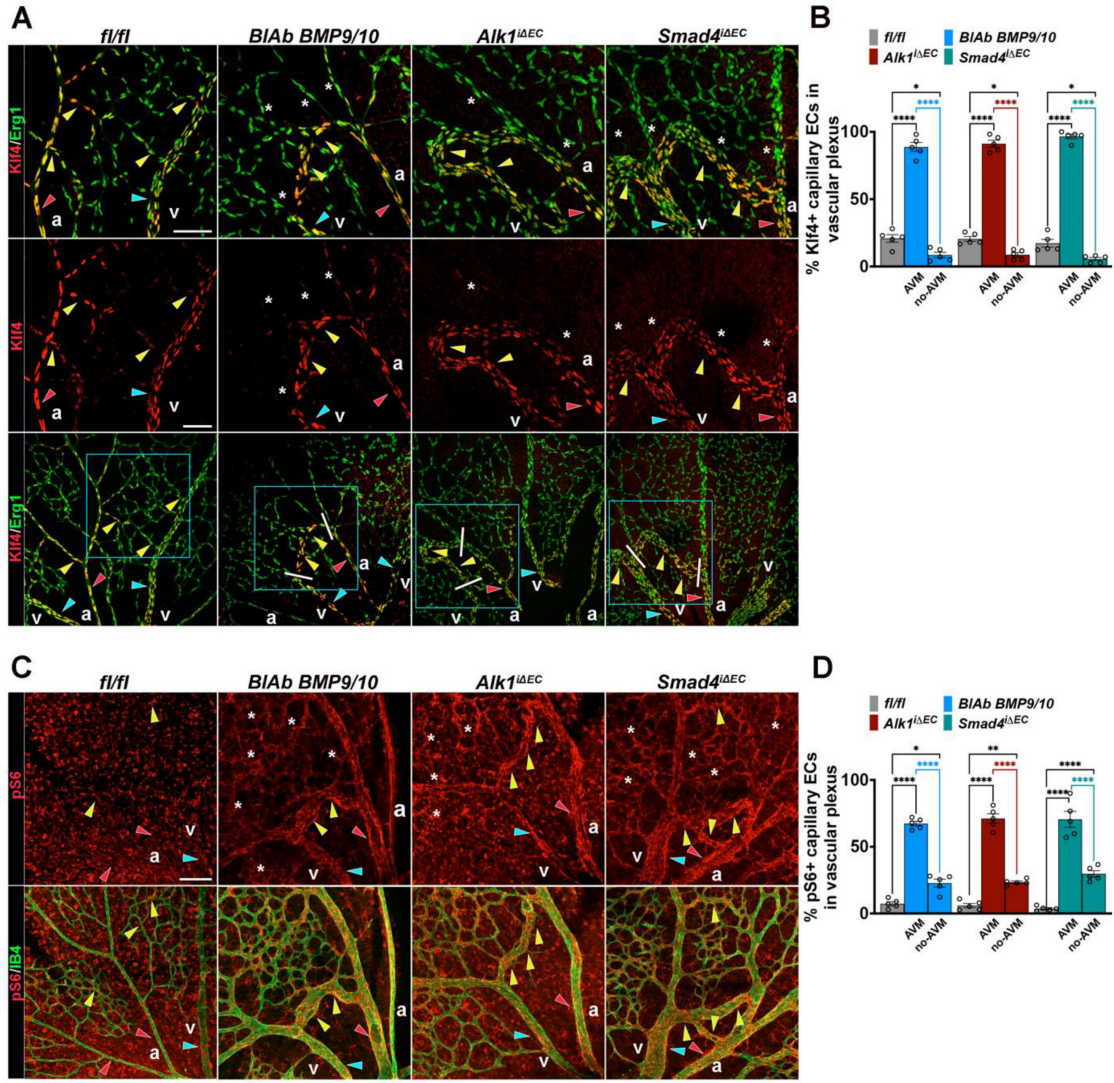
** Excessive Klf4-Akt signaling marks the retinal HHT-like AVMs. (A*,* C)** Images from P6 retina capillary plexus isolated from neonates treated with BMP9/10 BlAbs or tamoxifen-induced *fl/fl* , *Alk1*^iΔEC^, and* Smad4*^iΔEC^ stained for Klf4 (middle panel-red) and co-labeling for Klf4 and Erg1 (green) in lower and upper panels **(A)**; and for pS6 (red, upper panel) and co-labeling for pS6 (red) and IB4 (green) in the lower panel **(C)**. Red arrowheads indicate arteries, blue arrowheads indicate veins, yellow arrowheads indicate capillaries Klf4^+^ **(A)** and pS6^+^ **(C)**. Capillaries engaged in AVMs are considered the regions between the artery and the vein, demarked by white lines in the lower panel in **A**. White stars in BlAbs, *Alk1*^iΔEC^ and* Smad4*^iΔEC^ retinas indicate non-AVM capillaries Klf4^-^ **(A)** or pS6^+^ **(C)**. **(B, D)** Quantification of Klf4^+^ Erg1^+^ ECs (%) **(B**; n = 5 retinas/genotype from 3-5 mice/genotype**)** and of pS6 fluorescence labeling intensity **(D**; n = 5 retinas from 3-5 mice/genotype**)** in vascular plexus capillaries of *fl/fl*, AVM and non-AVM regions from the indicated genotypes. a, artery; v, vein. Scale bars: 50 μm **(A, C)**. Statistical significance was determined using 1-way Anova. Data are presented as mean ± SEM with adjusted p-values. *P < 0.05; **P < 0.01; ****P < 0.0001.

To validate these findings in murine models of HHT, we analysed retinas from Tx induced P6 Cre-negative fl/fl (control), BlAb BMP9/10 treated neonates, *Alk1^iΔEC^* and *Smad4^iΔEC^*. Retinas were immunolabeled for Klf4 (red) and Erg1 (green) (**Figure 2A**), and for IB4 (green) and pS6 (red), as a readout of activated Akt (**Figure 2C**).

In developing control retinas, Klf4 expression was high in major vessels and in ECs at capillary branch points exposed to shear stress, but nearly absent in low flow capillaries (**Figure 2A**, fl/fl). All mutant retinas, regardless of genotype displayed strong Klf4 expression in arteries, veins and capillaries forming AVMs (red, blue and yellow arrowheads), whereas non-AVM capillaries downregulated Klf4 (white stars in** Figure 2A**, quantified in** 2B**) consistent with lower flow outside of AVMs 34. In contrast, pS6 expression was elevated in all mutant capillaries (white stars in** Figure 2C**) and further enhanced in high-flow AVMs (yellow arrowheads in **Figure 2C**, quantified in **2D**). Together, these results demonstrate that excessive Klf4-Akt signaling characterizes the retinal HHT-like AVMs, independent of the underlying genetic defect.

### KLF4-Akt hyperactivation marks human AVM

**Figure 3:**
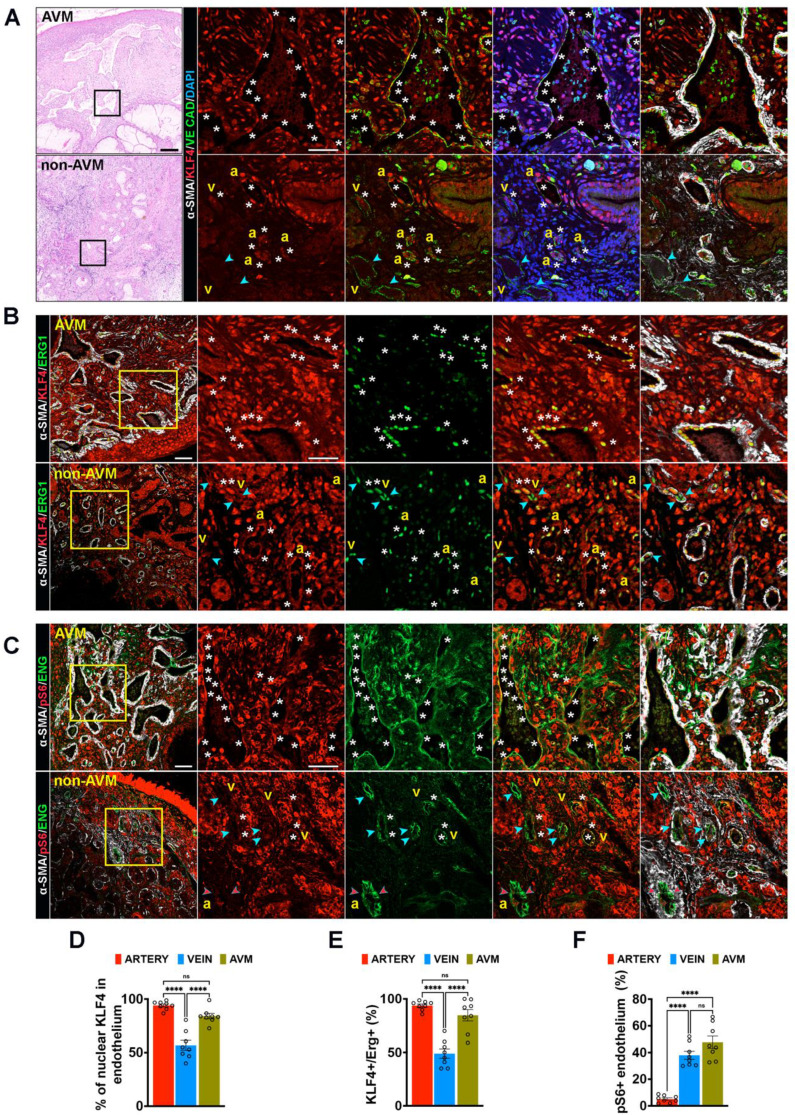
** Increased KLF4-Akt signalling marks the HHT2 telangiectasias. (A)** H&E staining of nasal mucosa biopsies sections from an HHT2 patient within the AVM region (left, upper panel) versus non-AVM region (left, lower panel). Insets show higher magnifications of sections immunolabeled for KLF4 (red), VE-Cadherin (green), α-SMA (white) and DAPI (blue). White stars indicate KLF4⁺ ECs; blue arrowheads indicate KLF4^-^ venous ECs. **(B)** Immunolabeling of HHT2 patient sections for KLF4 (red), ERG1 (green), and α-SMA (white) in AVM regions (upper panel) versus non-AVM regions (lower panel). White stars indicate KLF4⁺ ERG⁺ ECs; blue arrowheads indicate KLF4^-^ ERG1^+^ venous ECs. **(C)** Immunolabeling for pS6 (red), ENG (green), and α-SMA (white) in AVM regions (upper panel) versus non-AVM regions (lower panel). White stars indicate pS6⁺ ENG⁺ ECs; blue arrowheads indicate pS6^-^ ENG^+^ venous endothelium; red arrowheads indicate pS6^-^ ENG^+^ arterial endothelium. **(D)** Quantification of % of Klf4^+^ ECs in arterial and venous ECs in non-AVM, and within the AVM (n = 8 (4 pictures/patient biopsy). **(E)** Quantification of the number of KLF4^+^ ERG1^+^ ECs (%) (n = 8 (4 pictures/patient biopsy). **(F)** Quantification of pS6⁺ fluorescence labelling intensity (%) in ENG^+^ arterial versus venous endothelium, in non-AVM and AVM regions (n = 8 (4 pictures/patient biopsy). a, artery; v, vein. Scale bars: 200 μm in **A**, left panels; 100 μm in **B**, **C** left panels, 20 μm in insets in **A**, **B**, **C**. Statistical significance was determined using 1-way Anova. Data are presented as mean ± SEM with adjusted p-values. ns: non significant; ****P < 0.0001.

We next examined weather this mechanism is conserved in human AVMs from HHT2 patient biopsies. H&E staining revealed characteristic vascular abnormalities, including tortuous and dilated blood vessels in the nasal mucosa sections (**Figure 3A**, left panels). To assess EC activation, HHT2 samples were immunolabeled for KLF4 (red), VE-Cadherin (endothelial junctional marker, green), α-SMA (for SMC, to distinguish arterial from venous endothelium, white) and DAPI (for nuclei, blue) (**Figure 3A**, right panels). Comparison of KLF4 expression between AVM and the adjacent non-AVM regions from the same donor showed that most EC nuclei within the AVM were strongly KLF4 positive (white stars in upper right panels), whereas non-AVM regions displayed higher KLF4 expression predominantly in arterial ECs rather than in venous ECs (white stars versus blue arrowheads in lower right panels) (**Figure 3A**,**3D**). These results were corroborated by co-staining for KLF4 (red), α-SMA (white) and ERG1 (green) (**Figure 3B** upper versus lower panel, **3E**).

To validate increased Akt activation within the human AVMs, HHT2 samples were further labelled for pS6 (red), ENG (green) and α-SMA (white) (**Figure 3C**). In accordance with previous findings 35, 36, the AVM endothelium exhibited strong pS6 immunoreactivity (white stars in upper panels), while in non-AVM regions, pS6 expression was more pronounced in venous like endothelium (white stars) than in arterial ECs (red arrowheads in lower panels) (**Figure 3C**,**3F**). Collectively, these data demonstrate that overactivation of the Klf4-Akt signaling is a consistent feature of human HHT2 AVMs.

### Dysregulated EC proliferation as a cellular hallmark of AVMs

Enhanced EC proliferation triggers AVM formation in HHT murine models and pharmacological or genetical induction of cell cycle arrest, targeting Cdk4/6 has been shown to rescue this phenotype 12, 19, 20. To assess EC proliferation across different genotypes, we labelled retinas for 5-ethynyl-2′-deoxyuridine (EdU) to mark ECs in S-phase (green), Erg1 (white) and IB4 (red). An increased number of EdU^+^ ECs was detected within the AVMs, irrespective of the genotype (**Figure S1A**-**B**), confirming previous findings.

To validate these data in patients, HHT2 biopsy samples were immunolabelled for proliferating nuclear antigen (PCNA, red), α-SMA (white), ENG (green) and DAPI (blue).

**Figure 4:**
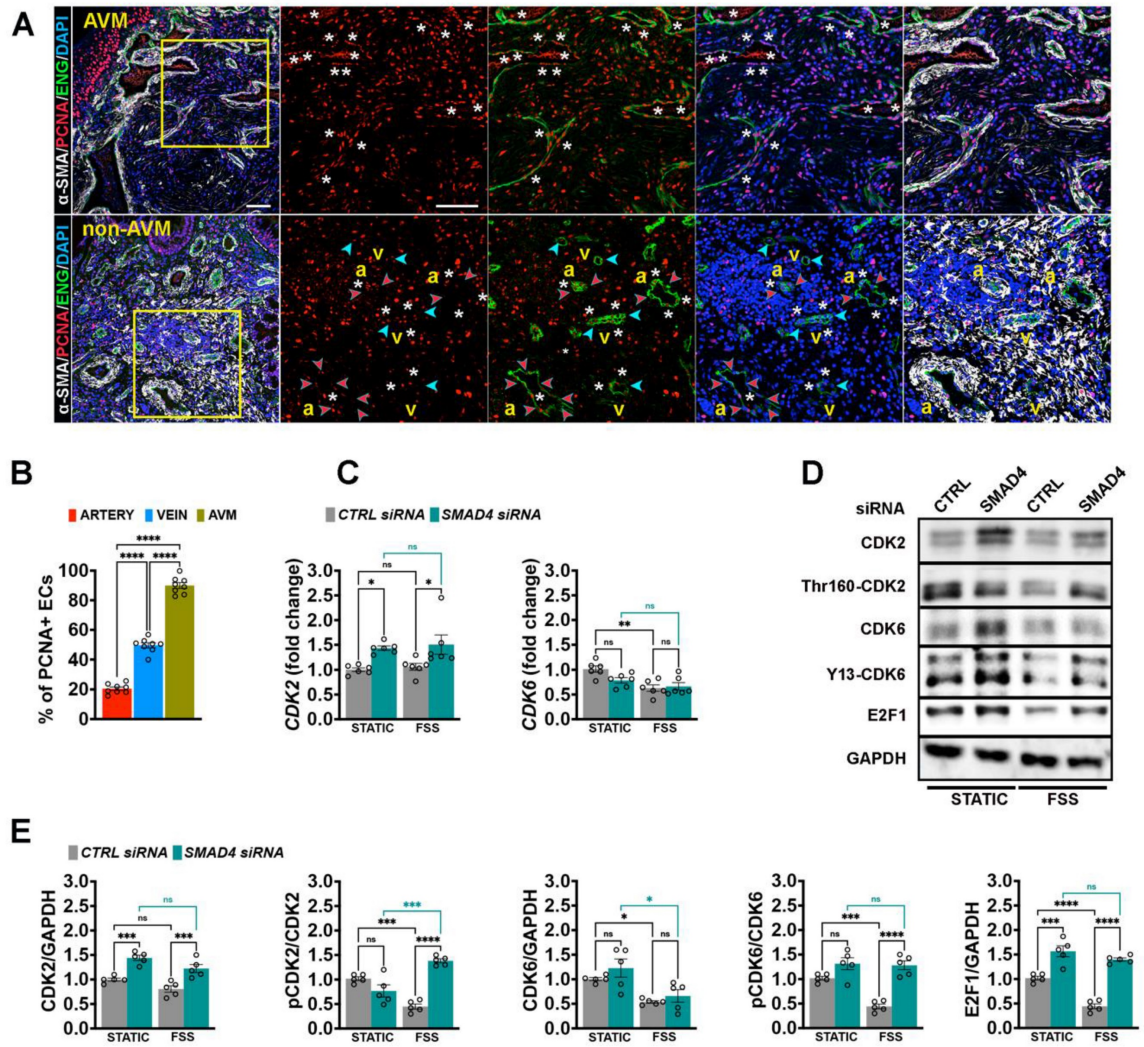
** Increased endothelial proliferation as a cellular hallmark of AVMs. (A)** Immunolabeling for PCNA (red), Endoglin (green), α-SMA (white) and DAPI (blue) in AVM region (upper panel) versus non-AVM region (lower panel). White stars define PCNA^+^ ENG^+^ ECs; blue and red arrowheads indicate the venous and arterial, respectively, ECs PCNA^-^. **(B)** Quantification of proliferating PCNA^+^ ECs in arterial versus venous ECs in non-AVM versus AVM regions (%), (n = 8, 4 pictures/patient biopsy). **(C)** qPCR for *CDK2* and *CDK6* in HUVECs transfected with *CTRL* and* SMAD4* siRNAs grown in static or subject to 12 Dynes/cm^2^ for 24 hours, (n = 6 independent experiments/group). **(D)** WB for CDK2, pCDK2, CDK6, pCDK6, E2F1 and GAPDH in HUVECs transfected with *CTRL* and *SMAD4* siRNAs grown in static or subject to 12 Dynes/cm^2^ for 24 hours. **(E)** Quantifications of pCDK2, pCDK6 and E2F1 levels normalized to total CDK2, CDK6 and GAPDH for the indicated conditions (n = 5 independent experiments/group). a, artery; v, vein. Scale Bars: 100 μm in left panel in **A**; 50 μm in insets. Statistical significance was determined using 1-way Anova in **B** and 2-way Anova with Tukey's multiple comparison test in **C**,** E**. Data are represented as mean ± SEM with adjusted p-values. ns: non-significant, *P < 0.05, **P < 0.01, ***P < 0.001, ****P < 0.0001.

Nearly all ECs ENG^+^ within the AVM exhibited strong PCNA expression (white stars in upper panels), while in non-AVM regions, PCNA positivity was largely confined to venous ECs (white stars versus blue and red arrowheads in lower panels, **Figure 4A**-**B**).

Mechanistically, we previously demonstrated that Smad4 is essential for flow-induced G1 arrest 12. To determine whether this mechanism extends to other HHT variants, we depleted *SMAD4*, *ALK1* or *ENG* in HUVECs, exposed the cells to physiological FSS for 24 h, and analyzed CDK-E2F pathway activation by qPCR (**Figure 4C**, **Figure S2A**-**B**) and WB (**Figure 4D**-**E**, **Figure S2C**-**F**).

Interestingly, depletion of *ALK1* or *ENG,* but not of *SMAD4*, abolished FSS-mediated repression of *CDK6*, at both mRNA and protein levels. Loss of *SMAD4* or *ENG* increased *CDK2* mRNA and protein expression, whereas *ALK1* knockdown (KD) or FSS exposure alone had no significant effect (**Figure 4C**-**E**, **Figure S2A**-**F**). Under control conditions, FSS reduced phosphorylation of CDK2 (Thr160-CDK2) and CDK6 (Y13-CDK6) and decreased E2F1 protein levels, consistent with FSS-induced cell cycle arrest. Strikingly, depletion of *SMAD4*, *ALK1* or *ENG* abolished FSS-mediated CDK2/6 inhibition and blocked the effect of FSS on downregulating E2F1. To note, *ALK1* depletion affected significantly only CDK6 activation (**Figure 4C**-**E**, **Figure S2C**-**F**). Together these results identify dysregulated EC proliferation as a shared cellular hallmark of AVMs and establish Smad4-CDK2/6-E2F axis as a critical mediator of flow dependent EC cycle control.

### Klf4-Akt overactivation in AVM disrupts PDGFB-PDGFRβ signaling

**Figure 5:**
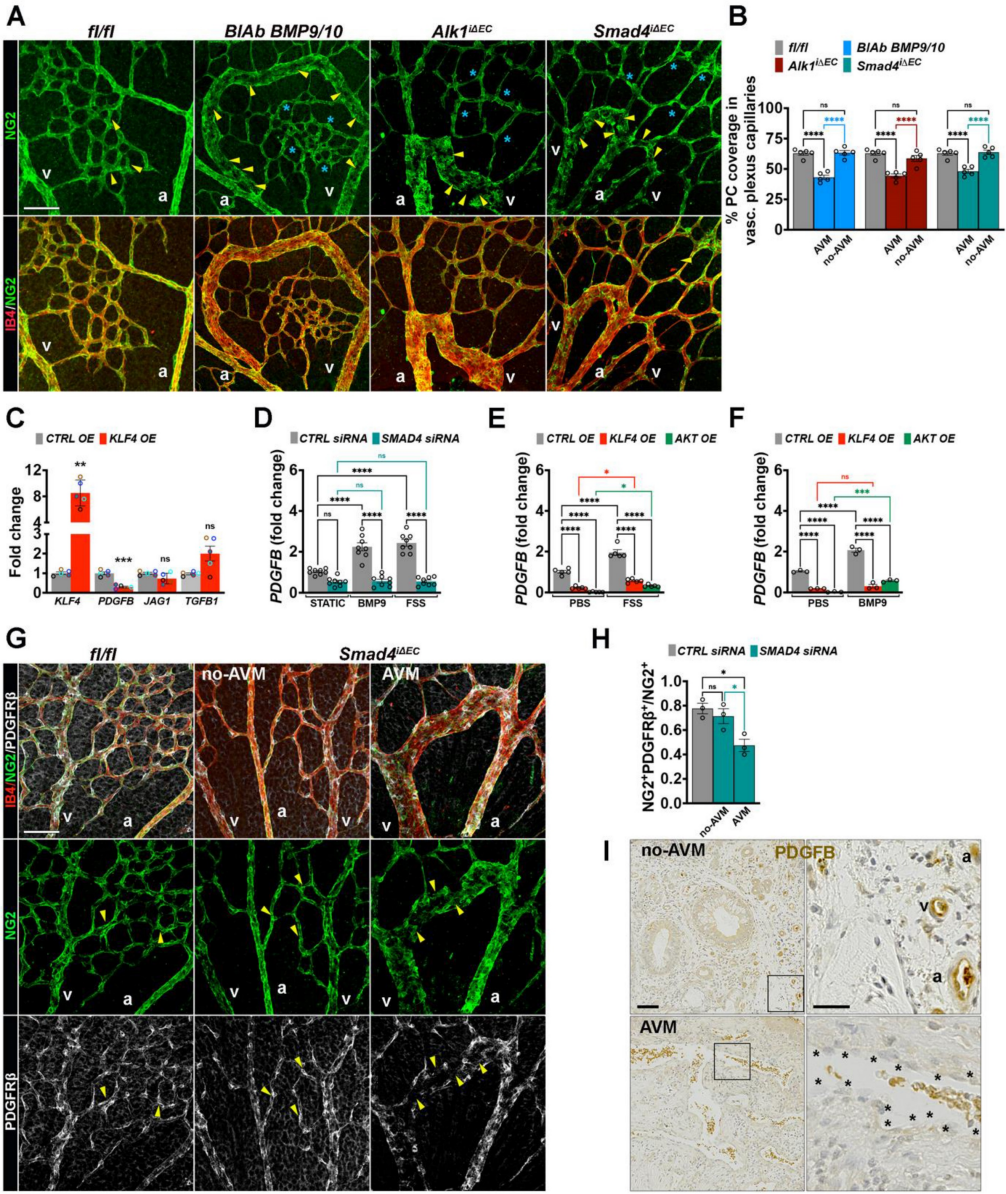
** Dysfunctional PDGFB-PDGFRβ leads to impaired pericyte association within AVMs. (A)** Representative images of the P6 retina capillary plexus from neonates treated with BMP9/BMP10 BlAb or Tx-induced* fl/fl*, *Alk1*^iΔEC^, and* Smad4*^iΔEC^ stained for NG2 (green, upper panel) and NG2 with IB4 (red) in lower panel. Yellow arrowheads indicate capillary ECs covered by NG2^+^ PCs in* fl/fl*, and AVM ECs lacking NG2^+^ PCs (NG2⁻); blue stars indicate non-AVM capillaries, NG2⁺. **(B)** Quantification of % of pericyte coverage in vascular plexus capillaries of indicated genotypes, (n = 5 retinas from 3-5 mice/genotype). **(C)** qPCR analysis of *KLF4*, *PDGFB*, *JAG1*, and* TGFb1* mRNA levels in HUVECs transduced with *KLF4 OE* or *CTRL OE* lentiviral constructs, (n = 5 per group). **(D)** *PDGFB* fold change in *SMAD4* and *CTRL* siRNA-transfected HUVECs grown in static conditions, or stimulated with 10 ng BMP9 or subject to 12 Dynes/cm² FSS for 4 h (n = 8 independent experiments per group). **(E,F)** *PDGFB* fold change in HUVECs transfected with *KLF4 OE, AKT OE* or *CTRL OE* lentiviruses and treated with 10 ng BMP9 for 4 h in static conditions (**E**, n = 5 per group) or subject to 12 Dynes/cm^2^ for 4 h (n = 3 per group) **(F)**.** (G)** Representative images of the P6 retina capillary plexus from Tx induced *Smad4 fl/fl* and* Smad4*^iΔEC^ neonates stained for IB4 (red), NG2 (green), and PDGFRβ (white). Yellow arrowheads indicate NG2⁺ PDGFRβ⁺ capillaries in *Smad4fl/fl* (left panel) and non-AVM regions (middle panel), and NG2⁺ PDGFRβ⁻ capillaries in AVM (right panel). **(H)** Quantification of the fraction of NG2^+^ PCs that expresses PDGFRβ in the vascular plexus capillaries of *Smad4^fl/fl^* retinas and of *Smad4*^iΔEC^ engaged or not engaged in AVMs, using the Manders overlap coefficient (n = 3 retinas from 3 mice/genotype). **(I)** Representative immunohistochemistry images for PDGFB staining in HHT2 patient biopsies, comparing non-AVM to AVM regions (1 biopsy/patient in 2 HHT2 patients). a, artery; v, vein. Scale Bars: 50 μm in **A**,**G**. 100 μm in left panels in **I** and 20 μm in insets. Statistical significance was determined using t-test in **C**, 1-way Anova in **B** and **H** and 2-way Anova with Tukey's multiple comparison test in **D**,**E**,**F**. Data are represented as mean ± SEM with adjusted p-values. ns: non-significant, *P < 0.05, **P < 0.01, ****P < 0.0001.

Beyond inducing EC cell cycle arrest, homeostatic FSS contributes to vessel stabilization by promoting mural cells recruitment through the endothelial regulation of chemoattractant factors. FSS modulates the expression genes such as *Pdgfb* (encoding PDGF-B), *Tgfb1* (encoding TGF-β1) and *Jag1* (encoding Jagged1) in ECs, which act in a paracrine manner to recruit PCs in an *Alk1*/*Eng* dependent fashion 4. To examine mural cell dynamics, we stained retinas from all genotypes for NG2 (green), a pericyte specific marker, together with IB4 (red). Regardless of genotype, AVMs were consistently characterized by a focal reduction in pericyte association (**Figure 5A**-**B**). Thus, the capillary Klf4^+^ Akt1^+^ cycling ECs within the AVM are deprived of PCs.

To explore the mechanistic link between overactivated Klf4-Akt signaling in endothelium and pericyte communication, we overexpressed *KLF4* in HUVECs using a lentiviral construct (*KLF4 OE*) and examined the expression of mural cell recruitment genes. Interestingly, *KLF4* overexpression led to a significant downregulation of *PDGFB* (**Figure 5C**). To further assess the regulation of *PDGFB* upon BMP9-FSS crosstalk, we stimulated* CTRL* versus* SMAD4* siRNA cells with BMP9, or subject them to laminar FSS. Both, BMP9 and FSS induced *PDGFB* expression in a *SMAD4* dependent manner (**Figure 5D**). Moreover, BMP9 and FSS stimulation failed to restore *PDGFB* expression in either *KLF4 OE* or *AKT OE* HUVECs (**Figure 5E**-**F**), suggesting that hyperactive KLF4-Akt overrides BMP9 and FSS-mediated upregulation of *PDGFB*.

To validate dysfunctional PDGFB signaling *in vivo*, retinas isolated from *Smad4^fl/fl^* and *Smad4*^iΔEC^ were labelled for IB4 (red), NG2 (green) and PDGFRβ (white). In *Smad4*^iΔEC^ retinas, the remaining NG2^+^ PCs within AVMs exhibited markedly reduced PDGFRβ expression (**Figure 5G**-**H**). Consistent with the data above, immunohistochemical analysis of HHT2 patient biopsies revealed pronounced downregulation of PDGFB specifically within the AVM endothelium compared with adjacent non-AVM vessels which retained PDGFB expression (**Figure 5I**). Together, these data demonstrate that AVMs are characterized by disrupted endothelial-pericyte communication via the PDGFB-PDGFRβ signaling axis and loss of pericyte identity.

### SMC gain characterizes the retinal murine HHT-like AVM and human telangiectasias

**Figure 6:**
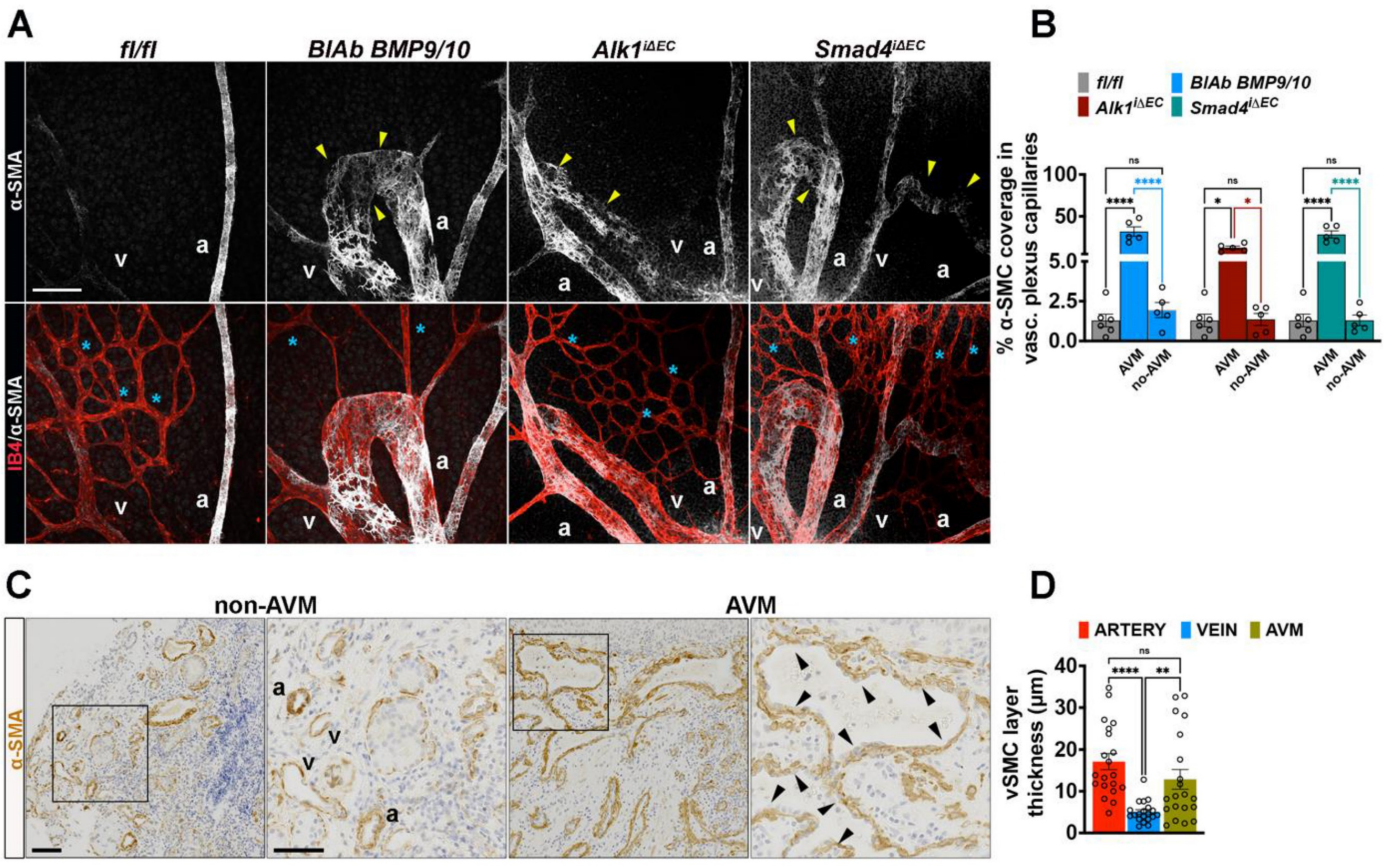
** AVMs are characterized by gain of SMCs. (A)** Representative images of the P6 retina capillary plexus from neonates treated with BMP9/BMP10 BlAb or Tx induced fl/fl, *Alk1*^iΔEC^ and* Smad4*^iΔEC^ stained for α-SMA for the vascular SMCs (white, upper panels), and co-labeling for α-SMA and IB4 (red) in lower panels. Yellow arrowheads indicate α-SMA^+^ in AVM regions and blue stars indicate capillaries lacking α-SMA (α-SMA⁻) in all indicated genotypes. **(B)** Quantification of percentage of vascular SMC coverage in vascular plexus capillaries of indicate genotypes (n = 5 retinas from 3-5 mice/genotype). **(C)** Labelling of HHT2 nasal mucosa biopsy sections for α-SMA in non-AVM versus AVM regions. **(D)** Quantification of α-SMA^+^ layer thickness (μm) along the vessel length in non-AVM versus AVM regions, n = 20 measurements (10 measurements per arteriole or venule in non-AVM regions and 10 measurements per AVM per patient biopsy) in 2 HHT2 patients. a, artery; v, vein. Scale Bars: 50 μm in **A**. 100 μm in left panels in **C**, 50 μm in insets. 1-way Anova in **B**,**D** was used to determine statistical significance. Data are represented as mean ± SEM with adjusted p-values. ns: non-significant, **P < 0.01, ****P < 0.0001.

In contrast to pericyte coverage reduction, staining for α-SMA (white) and IB4 (red) revealed a pronounced gain of α-SMA (α-SMA^+^) within the capillaries engaged in AVMs (**Figure 6A**-**B**). Increased α-SMA^+^ mural cell coverage was also observed in human HHT2 patient nasal biopsies, where the entire AVM lesion was surrounded by a thick α-SMA^+^ layer (**Figure 6C**-**D**), highlighting mural cell remodeling as a hallmark of HHT associated vascular malformations.

### FSS-mediated Klf4-Akt overactivation impairs pericyte migration and mural cell remodeling *in vitro*

**Figure 7:**
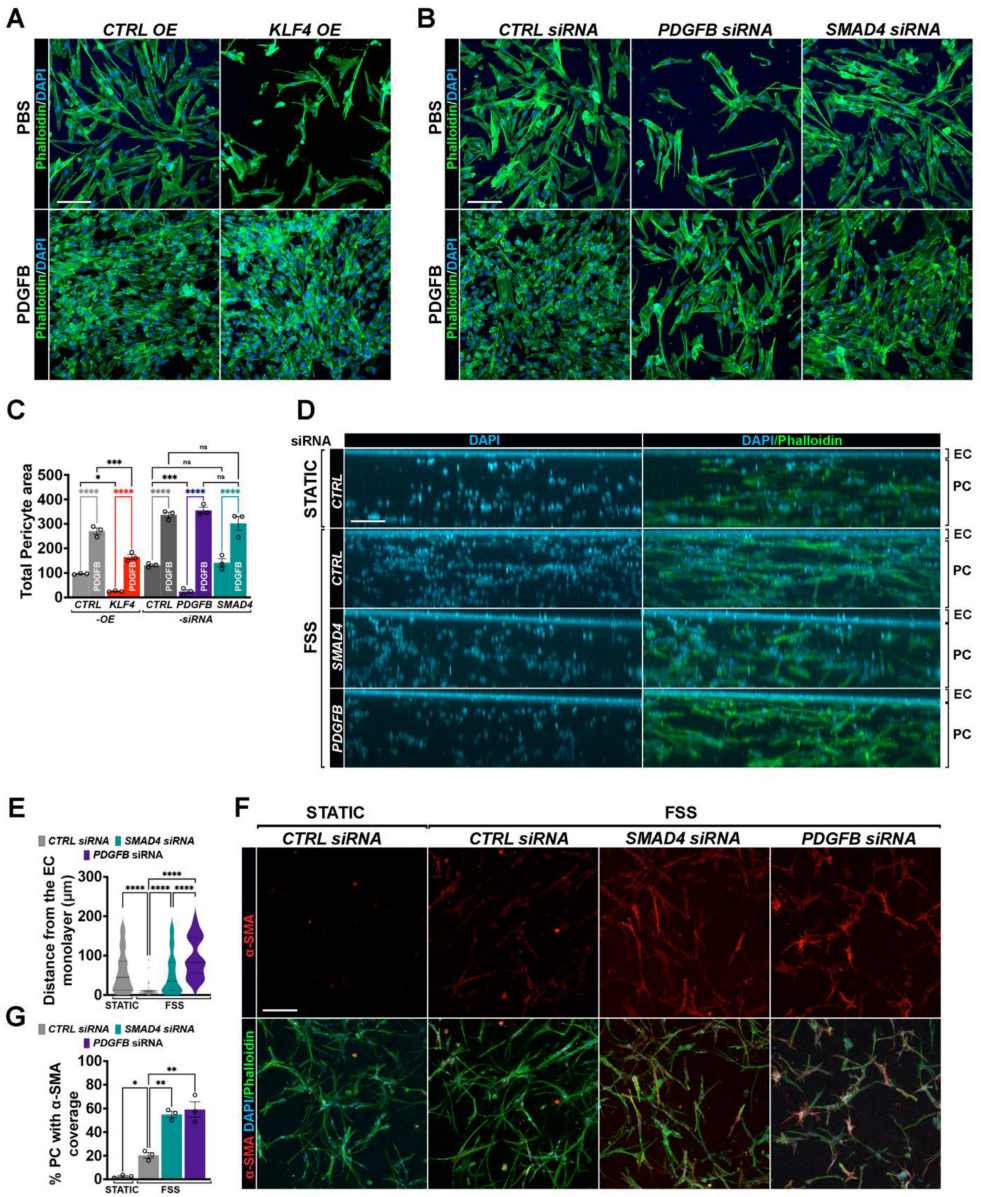
** Flow induced excessive Klf4-Akt signaling disrupts mural cell recruitment and remodelling. (A,B)** Phalloidin (green) and Dapi (blue) staining of migrated PCs towards HUVECs transduced with *CTRL* versus *KLF4* OE lentiviruses **(A)** or in *CTRL, PDGFB* and *SMAD4* siRNAs HUVECs **(B)** grown in 10% FCS medium and treated with 100 ng PDGFB (lower panels) versus PBS (upper panels) for 24 h. **(C)** Quantification of total pericyte area in the indicated genotypes (n = 3 independent experiments/genotype). (**D**,**F**) Representative images of YZ **(D)** and of XY **(F)** projections of a 200-μm thick stack from fibrin gels containing PCs and on top a monolayer of HUVECs transfected with *CTRL*, *SMAD4* and *PDGFB* siRNAs subject to 12 Dynes/cm^2^ FSS for 48 h in 10% FCS medium and stained with Phalloidin (green), and DAPI (blue) **(D)** and colocalization with α-SMA (red) **(F)**. **(E)** Relative migratory distance (μm) of PCs from the EC monolayer in static (*CTRL* HUVECs) or 48 h 12dynes/cm2 FSS conditions in CTRL, SMAD4 and PDGFB depleted HUVECs, n = 50 cells (average values of 50 pericyte migratory distance from the EC monolayer in 3 independent experiments/condition). **(G)** Quantification of % of PCs with α-SMA fluorescence in the indicated conditions (n = 3 independent experiments/condition). Scale Bars: 50μm in **A**,**B**,**F**; 100μm in **D**. Statistical significance was determined using 1-way Anova in **C**,**E**,**F**. Data are represented as mean ± SEM with adjusted p-values. ns: non-significant, *P < 0.05, **P < 0.01, ***P < 0.001, ****P < 0.0001.

We next evaluated whether aberrant endothelial Klf4-Akt signaling impairs pericyte migration using a transwell chemotactic migration assay with PCs labelled for phalloidin (green) and DAPI (blue). Similar to the effect observed with *PDGFB* siRNA, pericyte migration towards ECs was markedly reduced when HUVECs overexpressed *KLF4* or *AKT* (**Figure 7A**,**C**; **Figure S3A**-**B**). Addition of exogenous PDGFB fully restored pericyte migration, confirming that the inhibitory effect of Klf4-Akt activation is mediated through reduced endothelial PDGFB signaling. In contrast, *SMAD4*KD did not significantly alter pericyte migration in the absence of flow (**Figure 7B**-**C**). To assess the functional relevance of the flow-mediated effects, we employed a three-dimensional co-culture system, in which *CTRL* versus HUVECs depleted for* SMAD4* or* PDGFB* were plated on top of fibrin gels containing PCs, and flow was applied over the EC layer for 48 h. FSS promoted migration of the PCs toward the *CTRL* endothelial monolayer when compared to static conditions (**Figure 7D**-**E**). In this setting, ECs with *SMAD4*KD, similar to* PDGFB* depletion, abolished pericyte migration toward the endothelial layer when compared to *CTRL* siRNA HUVECs (**Figure 7D**-**E**), supporting the notion that flow- induction of endothelial PDGFB signaling is Smad4 dependent. In the same experimental set-up, flow promoted a slight increase in α-SMA expression in PCs co-cultured with control ECs, reflecting physiological mural cell remodeling (**Figure 7F**-**G**), an effect further exacerbated upon loss of EC *SMAD4* or *PDGFB*. Together, these findings show that Smad4 is required to maintain physiological PDGFB-mediated endothelium-pericyte communication during FSS-driven mural cell recruitment and remodeling.

### Active EC cycling downstream of excessive Klf4-Akt regulates* PDGFB* expression

**Figure 8:**
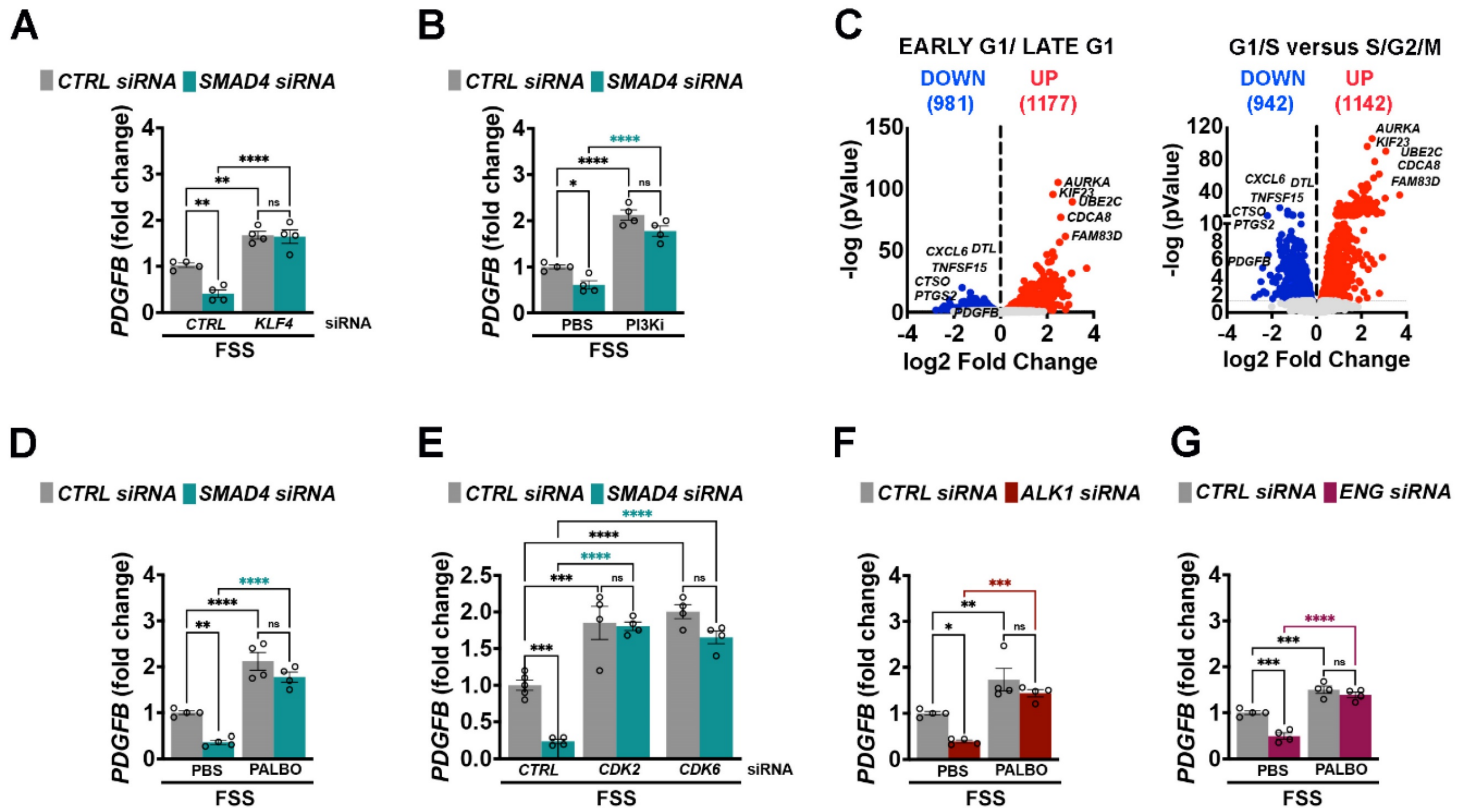
** KLF4-Akt signaling mediated cell cycle arrest regulates *PDGFB* expression. (A,B,D,E)** *PDGFB* fold change in HUVECs grown in complete medium and subject to 24 h 12 Dynes/cm^2^ FSS transfected with *CTRL*, *SMAD4*, *KLF4* and combined *SMAD4*;*KLF4* siRNAs (n = 4 independent experiments /group) **(A)**, with *CTRL* and *SMAD4* siRNAs HUVECs treated with PBS versus PI3K inhibitor (Pictilisib, 20nM) for 24 h (n = 4 independent experiments/group) **(B)**, with *CTRL* and *SMAD4* siRNAs HUVECs treated with PBS or 2 μM Palbociclib for 24 h (n = 4 independent experiments/group) **(D)**, with *CTRL*, *SMAD4*, *CDK2*, *CDK6* and combined *SMAD4*;*CDK2* and *SMAD4*;*CDK6* siRNAs (n = 4 independent experiments/group) **(E)**. **(C)** Volcano plots of fold-change against the log 10 (p-value) in HUVECs-FUCCI reporter line that distinguishes gene differentially expressed in early G1 and late G1 cell cycle states (left) and in G1/S versus S/G2/M (right). (**F**,**G**) *PDGFB* fold change in HUVECs subject to 24 h 12 Dynes/cm^2^ transfected with *CTRL* and *ALK1* siRNAs **(F)** or *CTRL* and *ENG* siRNAs **(G)** treated with 2 μM Palbociclib for 24 h (n = 4 independent experiments/group). 1-way Anova was used to determine statistical significance. Data are represented as mean ± SEM with adjusted p values. ns: non-significant, *P < 0.05, **P < 0.01, ***P < 0.001, ****P < 0.0001.

As genetical or pharmaceutical inhibition of Klf4-PI3K/Akt-Cdk2/6 axis rescues AVM formation 12, 19, 20, we next examined whether this pathway regulates *PDGFB* expression. In contrast to *KLF4* and *AKT* gain of function (GOF) approaches which suppressed *PDGFB*, inactivation of *KLF4* using *KLF4* siRNA or inhibition of PI3K/Akt activation using Pictilisib, a specific PI3K inhibitor, restored the FSS-mediated *PDGFB* expression in *SMAD4*KD cells (**Figure 8A**-**B**).

To determine whether *PDGFB* regulation depends on cell cycle status, we re-analysed previous published bulk RNA sequencing data from HUVECs expressing the Fast-FUCCI reporter 37. Notably, *PDGFB* expression was significant reduced in ECs transitioning from G1/S towards S/G2/M (**Figure 8C**-**D**), suggesting that excessive cell cycle progression represses *PDGFB* expression. To experimentally validate this link, *CTRL* and *SMAD4* siRNAs HUVECs subjected to physiological FSS were treated with Palbociclib, a specific CDK4/6 inhibitor. Reinforcement of cell cycle arrest potently induced *PDGFB* expression in both *CTRL* and* SMAD4KD* cells (**Figure 8D**). Similarly, depletion of *CDK2* or *CDK6* induced *PDGFB* levels (**Figure 8E**), and Palbociclib treatment rescued *PDGFB* expression in *ALK1* and* ENG* knockdown cells as well (**Figure 8F**-**G**). Thus, taken together, these data indicate that Alk1/Eng-Smad4 signaling is required for FSS-mediated CDK2/6 inactivation, thereby maintaining cell cycle arrest and PDGFB expression.

### Thalidomide mediated PDGFB upregulation rescues AVM formation

**Figure 9:**
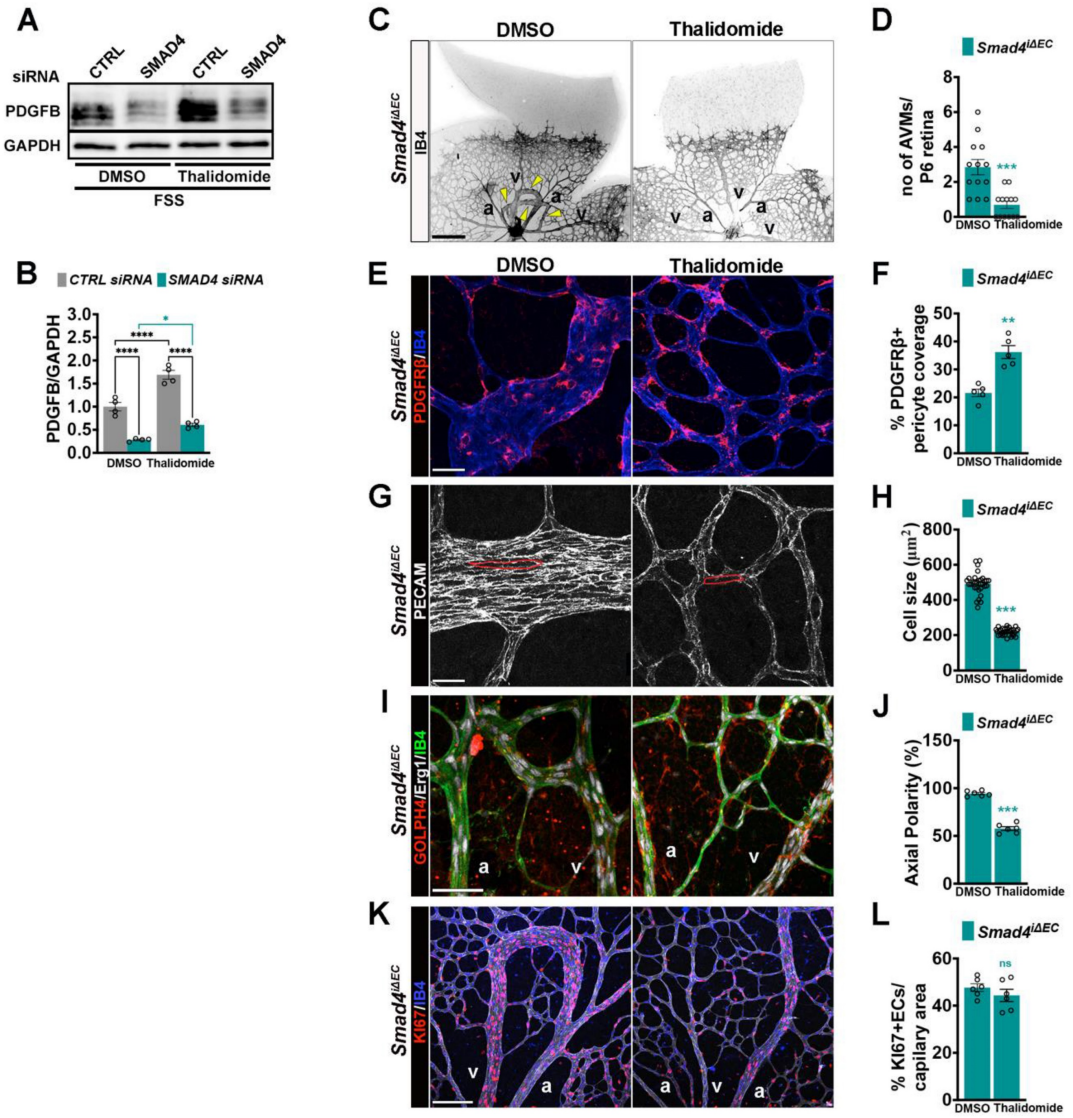
** Thalidomide induced PDGFB expression improves AVM formation. (A)** WB for PDGFB and GAPDH in *CTRL* and *SMAD4* siRNAs HUVECs grown in complete medium and subject to 12 Dynes/cm^2^ FSS for 24 h treated with DMSO or thalidomide (100 μM). **(B)** Quantification of PDGFB protein levels normalized to GAPDH in the indicate conditions (n = 4 independent experiments/group). **(C)** Negative confocal images of IB4 stained of P6 retinas from Tx induced *Smad4*^iΔEC^ neonates treated with DMSO or thalidomide. Yellow arrowheads indicate AVMs. **(D)** Quantification of AVM number per P6 retina in DMSO and thalidomide treated *Smad4*^iΔEC^ neonates (n = 13 retinas from 7-8 mice/genotype). **(E,G,I,K)** High magnification confocal images of P6 retina vascular plexus from Tx induced *Smad4*^iΔEC^ DMSO or thalidomide treated neonates, labeled for PDGFRβ (red) and IB4 (blue) **E**; for PECAM (white) **(G)**; for GM130 (red), Erg1 (white) and IB4 (green) **(I)** and for KI67 (red) and IB4 (blue) **(K)**. **(F, H, J, L)** Quantification of % PDGFRΒ+ pericyte coverage per capillary area from images in E (n = 5 retinas from 3-5 mice/group) **(F)**; of EC size (μm^2^) from images in G (n = 30 (10 measurements per retina from 3 mice/group)) **(H)**; of % of oriented ECs against the direction of flow in vascular plexus capillaries from images in I (n = 6 (2 images per retina from 3 mice/group)) **(J)** and quantification of KI67^+^ ECs per vascular area from images in K **(L)** (n = 6 (2 images per retina from 3 mice/group)). a, artery; v, vein. Scale Bars: 100 μm in panel **A**, 20 μm in panels** E**,**G**,** I**; 50 μm in panel** K**. Statistical significance was determined using 2-way Anova with Tukey's multiple comparison test in **B** and t-test in **D**,**F**,**H**,**J**,**L**. Data are represented as mean ± SEM with adjusted p values. ns: non-significant, *P < 0.05, **P < 0.01, ***P < 0.001, ****P < 0.0001.

PDGFB is required for pericyte recruitment and maintenance on the developing endothelium layer 5, 6. Thalidomide has been shown to enhance PDGFB expression restoring mural coverage and vessel integrity in murine and human HHT lesions 30-33. To validate previous findings, we tested thalidomide effect on FSS-induced *PDGFB* expression regulation in *CTRL* and *SMAD4* siRNA HUVECs. Interestingly, thalidomide further increased PDGFB expression upon flow in *CTRL* but also in *SMAD4*KD cells (**Figure 9A**-**B**), suggesting that the thalidomide effect on PDGFB regulation is Smad4 independent. To explore whether PDGFB induction ameliorates AVMs, we treated Tx induced *Smad4*^iΔEC^ neonates with thalidomide or DMSO. Thalidomide treatment significantly reduced AVM number (**Figure 9C**-**D**) and increased pericyte coverage (**Figure 9E**-**F**). Thalidomide treatment also normalized EC size (**Figure 9G**-**H**) and orientation against flow (**Figure 9I**-**J**), while the EC proliferation was not significantly inhibited (**Figure 9K**-**L**). These findings demonstrate that restoration of PDGFB expression rescues multiple endothelial and mural cell defects, reinforcing PDGFB upregulation as a promising therapeutic strategy for HHT-associated AVMs.

## Discussion

Physiological shear stress promotes and maintains vascular homeostasis by coordinating endothelial behavior and endothelial-mural cell communication, but how these FSS-driven processes intersect under pathological flow conditions such as AVMs remains unknown. Loss of Smad4 signaling in HHT disrupts endothelial mechanotransduction, rendering ECs hyperresponsive to flow. We recently showed that this loss lifts BMP9-mediated repression of flow-induced Klf4 and the downstream Akt, resulting in excessive Klf4-Akt activation. This maladaptive activation disrupts FSS-induced G1 arrest 12, a process essential for arterial fate specification 11, 37. Our findings here extend previous observations in Smad4-deficient mice, by demonstrating that aberrant retinal Klf4-Akt activation is a convergent phenotype across multiple HHT genotypes, including *Alk1* or BMP9/10 ligand inhibition. The consistency of this signature across murine models and in human HHT2 telangiectasias suggests that dysregulated Klf4-Akt signaling acts as a common mechanistic hub integrating genetic and hemodynamic insults in HHT. Whether AVMs in visceral organs or in HHT1 models and patients arise via identical pathological mechanisms, remains to be further established.

While defects in EC polarity and migration have been implicated in AVM formation upon *Eng* or *Alk1* loss 16-18, mounting evidence supports aberrant EC proliferation as the principal driver of lesion formation across murine models 21. Our data extend these findings by showing that loss of SMAD4-ALK1/ENG blocks FSS-induced CDK2/6 inhibition, preventing repression of the E2F1 transcriptional program and thereby sustaining EC proliferation. How exactly this mechanism is activated in AVMs, which dysregulated cyclins or CDK-Cyclin complexes and which inhibitory CDKs are required, remain to be further investigated. Interestingly, this persistent cycling state correlates with transcriptional downregulation of *PDGFB*, encoding an essential paracrine signal for pericyte recruitment. The observation that PDGFB expression decreases during the G1 to S transition supports a model in which excessive cell-cycle progression limits pericyte supportive signaling. Thus, endothelial hyperproliferation is not merely a byproduct of AVM pathology, but a driver of pericyte detachment and vessel destabilization. Thus, our findings identify a flow-sensitive link between endothelial cell-cycle control and pericyte recruitment, emphasizing that flow-mediated EC cycle arrest is required not only for arterial specification but also for establishing stable EC - pericyte interface.

Our study also highlights mural cell remodeling as a consistent feature of HHT-associated AVMs with focal pericyte loss together with an abnormal enrichment of α-SMA⁺ SMCs. This phenotypic shift likely reflects a FSS-induced compensatory response to reduced pericyte coverage. Endothelial specific *Pdgfb* deficient neonatal retinas develop dilated, pericyte-depleted capillaries resembling AVM-like structures, yet with moderate ectopic α-SMA⁺ investment 5. Thus, focal suppression of PDGFB within the AVM endothelium likely accounts for impaired pericyte recruitment, whereas the increased SMC-like population, potentially arising through pericyte differentiation, may reflect dysregulation of additional FSS-responsive angiocrine signals. Interestingly, sporadic brain AVMs are typically characterized by reduced mural investment including both, PCs and SMCs 38. This difference likely reflects organ specific remodeling programs. During coronary artery development, angiocrine-paracrine Jagged1-Notch signaling promotes pericyte to SMC differentiation, an event initiated by the onset of blood flow 39. Notably, Jagged1 expression is reduced in AVM endothelium 23, 24, 40, and loss of canonical Notch signaling in PCs leads to AVM formation 41, highlighting dysfunctional Jagged1-Notch signaling at the endothelial-mural interface as a putative contributor to abnormal mural cell remodeling within the AVMs. Collectively, these observations delineate a two-step mechanism of AVM pathogenesis: (i) loss of BMP9/10 signaling leads to FSS mediated Klf4-Akt-Cdk-driven EC hyperproliferation, and (ii) this cycling state suppresses PDGFB production, promoting pericyte detachment and FSS-mediated aberrant mural cell remodeling.

PDGFB was among the first genes identified as shear-stress responsive 42, and its expression is modulated by Foxo1 43, which is inactivated by excessive Akt signaling in venous malformations 44. Akt inhibition reactivates Foxo1 24 and restores PDGFB levels in SMAD4-depleted ECs, indicating a shared disrupted pathway controlling PDGFB. Sox17, an arterial marker, also regulates PDGFB 45, putatively linking arterial identity to its expression. How BMP9 regulates PDGFB remains unknown and warrants further study.

Pharmacological restoration of PDGFB expression through thalidomide treatment rescues AVM formation, increases PC coverage, and restores EC morphology, though EC proliferation remained elevated. Thalidomide and its analogues (lenalidomide, pomalidomide) exert their effects primarily via binding to the E3 ubiquitin ligase substrate receptor Cereblon (CRBN) 46, thereby altering the stability of key transcriptional regulators. In ECs, thalidomide's anti-angiogenic properties have been linked to suppression of pro-angiogenic mediators including VEGF and ANGPT2 47, 48. Given that ANGPT2-Tie2 signaling acts upstream of Akt activation and Tie2 is transcriptionally regulated by Klf4 12, 49, these observations raise the possibility that thalidomide indirectly attenuates the Klf4-Akt pathway via modulation of CRBN-dependent transcriptional programs, while simultaneously promoting restoration of pericyte recruitment through enhanced PDGFB expression.

Future studies should explore whether combining thalidomide derivatives with CDK4/6 inhibitors, PI3K/Akt modulators, or direct PDGFB mimetics could yield synergistic therapeutic benefits in restoring vessel stability in HHT patients.

This study has several limitations. Firstly, our analyses were largely confined to retinal AVMs in neonatal mice and nasal HHT2 telangiectasis. Future investigations should capture organ-specific AVM heterogeneity, such as the brain, liver or lung. Additionally, while PDGFB emerged as a key downstream effector, other flow-regulated angiocrine pathways likely contribute to mural cell remodeling.

In conclusion, in the present study, we demonstrate that excessive KLF4-Akt-CDK signaling uncouples shear stress sensing mediated cell cycle regulation from PDGFB production, thereby driving pericyte loss and mural cell remodeling in AVMs. This establishes endothelial cell cycle state as the upstream determinant of mural cell stability under pathological flow. Beyond its implications for HHT, this pathway may represent a broader paradigm for understanding how dysregulated endothelial mechanotransduction leads to vascular anomalies.

## Supplementary Material

Supplementary figures and tables.

## Abbreviations

AVMs: Arteriovenous malformations
BMP: Bone morphogenic protein
LOF: Loss of function
HHT: Hereditary Hemorrhagic Telangiectasia
ECs: Endothelial cells
FSS: Fluid shear stress
PCs: Pericytes
SMCs: Smooth muscle cells
ALK1: Activin like kinase 1
ENG: Endoglin
SMAD4: SMAD, Mothers Against DPP Homolog 4
JP-HHT: Juvenile polyposis - Hereditary Hemorrhagic Telangiectasia
HUVECs: Human umbilical venous endothelial cells
Cdh5: Cadherin 5
fl/fl: flox/flox
P6: postnatal day 6
BlAb: Blocking antibodies
Tx: Tamoxifen
pSMAD1/5: phosphorylated SMAD1/5
Klf2: Krüppel-Like Factor 2
Klf4: Krüppel-Like Factor 4
Akt: Serine-Threonine Protein Kinase
Cdk2/6: Cyclin-dependent kinases 2/6
NG2: neural/glial antigen 2
α-SMA: alpha smooth muscle actin
PDGFB: Platelet-derived growth factor
PDGFRβ: Platelet-derived growth factor receptor β
PTEN: Phosphate And Tensin Homolog
PI3K: Phosphatidylinositol-4,5-Biphosphate 3-Kinase
pS6: phosphorylated ribosomal protein S6
IB4: Isolectin B4
α-SMA: Alpha smooth muscle actin
PCNA: Proliferating nuclear antigen
GOF: Gain of function
CTRL: Control
KD: Knockdown
Foxo1: Forkhead Box Protein O1
VEGF: Vascular Endothelial Growth Factor
ANGPT2: Angiopoietin-2
Tie2: Tyrosine-Protein Kinase Receptor, TEK
CRBN: Cereblon
rpm: rotation per minute
rt: room temperature
h: hours
U: unit
µm: micrometer
nM: nanomolar
µM: micromolar
WB: western blot
qPCR: quantitative polymerase chain reaction
TGF-β1: Transforming growth factor β1
Erg1: Early growth response factor 1
EdU: 5-ethynyl-2′-deoxyuridine
i.p: Intraperitoneal
