# Supplementary material for: Flow-induced Klf4-Akt signaling links EC cycling to mural cell defects in arterial-venous malformations
Source: Theranostics. 2026 Feb 26;16(9):4905–22. doi: 10.7150/thno.121154 (PMC12964382; doi:10.7150/thno.121154)
Supplement: Supplementary file 1 — Supplementary figures and tables. [file thnov16p4905s1.pdf]

Supplementary Figures

Flow-induced Klf4-Akt signaling links EC cycling to mural cell defects in AVMs

Yanzhu Lin[1,2*], Zohrah Hashemi[1*], Qing Zhang[1*], Yuxi Di[1], Tanmaya Behera[1], Johannes Gahn[1], Kuheli Banerjee[1], Fan Wu[1], Kornelia Andorfer[3], Mahak Singhal[4], Caroline Seebauer[3,5], Roxana Ola[1#]

Supplementary Figure 1

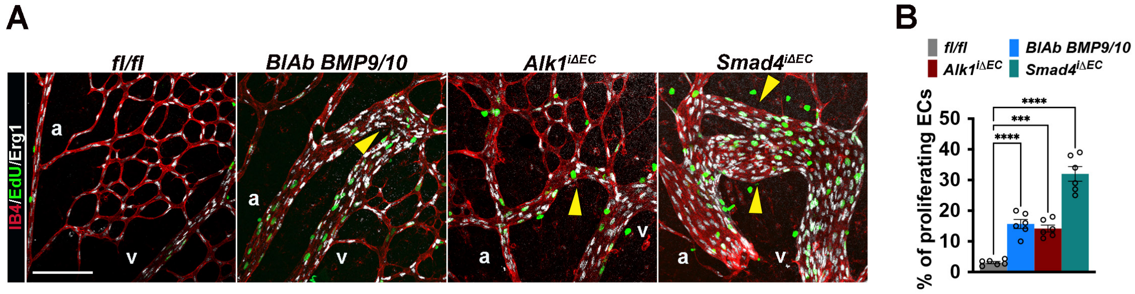

Supplementary Figure 1. Increased EC proliferation among cellular AVM hallmarks. (**A**) High magnification of representative images of P6 retina vascular plexus from neonates treated with BlAbs for BMP9/BMP10 or tamoxifen induced *Alk1*^{iΔEC} and *Smad4*^{iΔEC} versus non-Cdh5 Cre fl/fl labelled for EdU (green), Erg1 (white) and IB4 (red) after 4 h of intraperitoneal administration of EdU. Yellow arrowheads point to AVMs. (**C**) Quantification of proliferating EdU^{+} Erg^{+} ECs (%). a: artery; v: vein. Scale Bars: 50µm. t test in **B** was used to determine statistical significance. Data are represented as mean ± SEM with the adjusted p-values. ***P < 0.001 ****P < 0.0005.

Supplementary Figure 2

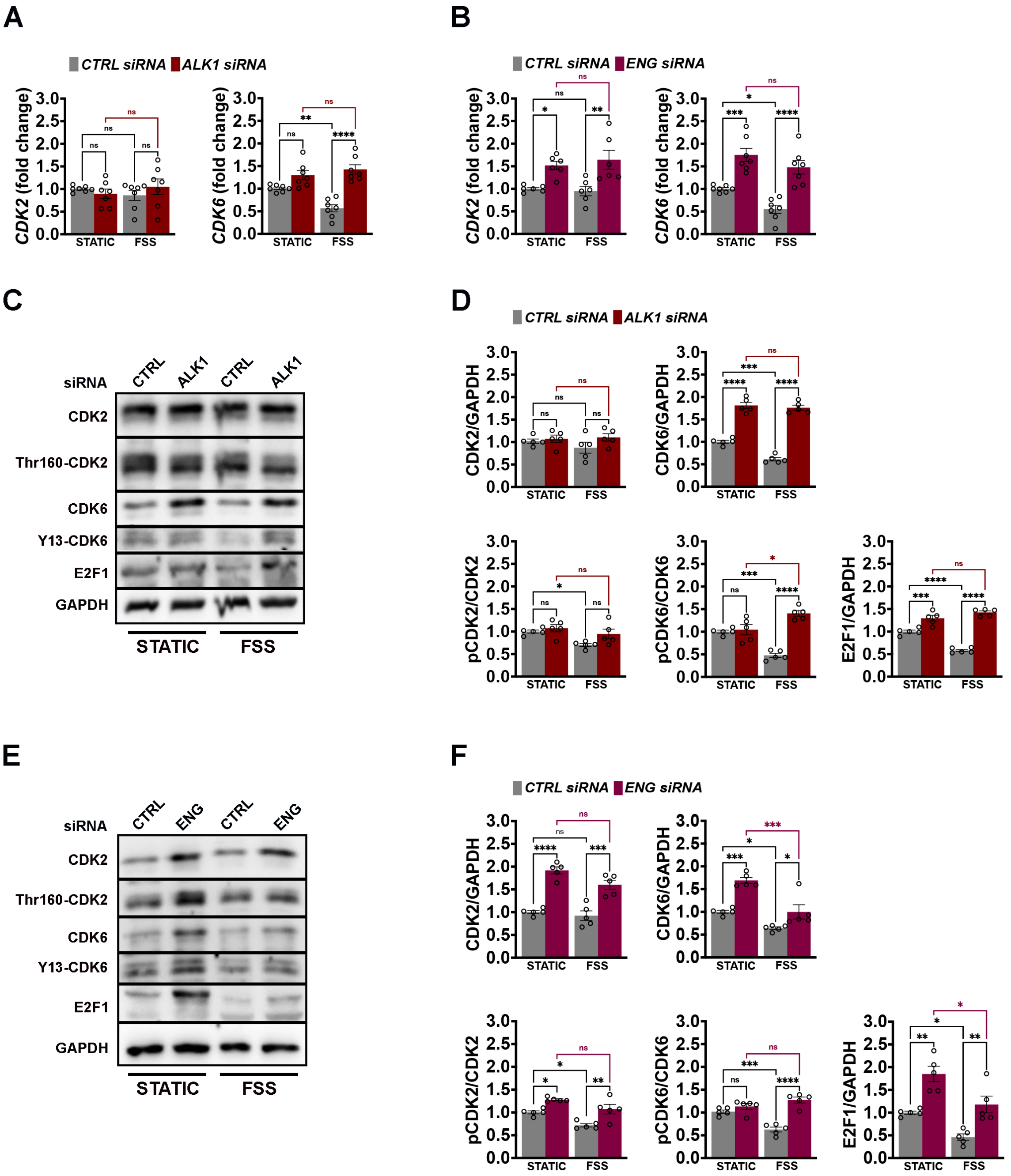

Supplementary Figure 2. Alk1-Eng signaling is required for FSS mediated cell cycle arrest. (**A**) qPCR for *CDK2* and *CDK6* in HUVECs transfected with *CTRL* and *ALK1* siRNAs grown in static or subject to 12 Dynes/cm^2 for 24 h (n=7 independent experiments/group). (**B**) qPCR for *CDK2* and *CDK6* in HUVECs transfected with *CTRL* and *ENG* siRNAs grown in static or subject to 12 Dynes/cm^2 for 24 h in complete medium, (n=6 independent experiments/group). (**C**,**E**) WB for CDK2, pCDK2, CDK6, pCDK6, E2F1 and GAPDH in HUVECs transfected with *CTRL* and *ALK1* siRNAs (**C**) and *CTRL* and *ENG* siRNAs (**E**) grown in static or subject to 12 dynes/cm^2 for 24 h in complete medium. (**D**,**F**) Quantifications of pCDK2, pCDK6 and E2F1 levels normalized to total CDK2, CDK6 and GAPDH for the

indicated conditions (n = 5 independent experiments/group). Statistical significance was determined using 2-way Anova in **A**, **B**, **D**, **F**. Data are represented as mean ± SEM with the adjusted p-values. ns: non-significant, $*P < 0.05$, $**P < 0.01$, $***P < 0.001$, $****P < 0.0001$

Supplementary Figure 3

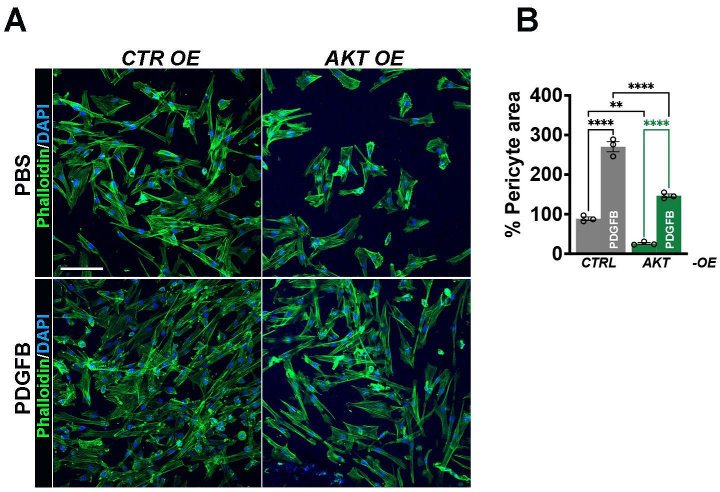

Supplementary Figure 3. Increased AKT leads to loss of pericyte migration.
(**A**) Phalloidin (green) and Dapi (blue) staining of migrated PCs towards HUVECs transduced with *CTRL* versus *AKT* OE lentiviruses grown in complete medium and treated with 100ng PDGFB (lower panels) versus PBS (upper panels) for 24 h. (**C**) Quantification of total pericyte area in the indicated genotypes (n=3 independent experiments/group). Scale Bars: 50µm in panel **A.** Statistical significance was determined using 1-way Anova in **B**. Data are represented as mean ± SEM with the adjusted p-values. ns: non-significant, $**P < 0.01$, $****P < 0.0001$.

Table S1. Primary antibodies for retina staining

Antibody	Dilution	Catalog Number, Company
Phosphorylated S6	1:100	5364S, Cell Signaling
Klf4	1:200	AF3158, R&D Systems
Erg	1:200	ab196149, Abcam
Isolectin B4 (IB4)	10 µg/ml	121412, Life Technologies
PECAM	1:1000	ThermoFisher
PDGFRß	1:100	AF1042, R&D Systems
NG2	1:200	Ab5320, Millipore
α-SMA	1:600	Ab184675, Abcam
Golph4	1:200	Ab28049, Abcam
KI67	1:500	50-5698-82, Thermo Fisher

Table S2. Primary antibodies for human patient staining

Antibody	Dilution	Catalog Number and Company
PCNA	1:200	13110S, Cell Signaling
α-SMA	1:250	M0851, Agilent
KLF4	1:50	AF3640, R&D systems
PDGFB	1:200	Ab23914, Abcam
Endoglin	1:100	AF1097, R&D systems
VE-Cadherin	1:25	2500S, Cell signaling
Phosphorylated S6	1:100	5364S, Cell Signaling
ERG1	1:100	Ab92523, Abcam
Phalloidin	1:1000	sc-363791

Table S3. Primary antibodies for WB

**Antibody**	**Dilution**	**Catalog Number and Company**
Endoglin	1:1000	AF1097, R&D
Akt	1:1000	4685, Cell Signaling
Phosphorylated Akt	1:1000	4060, Cell Signaling
SMAD4	1:1000	38454, Cell Signaling
ALK1	1:1000	70R-49334, Fritzgerald
Phosphorylated SMAD1/5	1:1000	9516S, Cell Signaling
SMAD5	1:1000	12534S, Cell Signaling
GAPDH	1:1000	5174, Cell Signaling
CDK2	1:1000	2546, Cell Signaling
pCDK2	1:1000	2561, Cell Signaling
CDK6	1:1000	3136, Cell Signaling
pCDK6	1:1000	ab131439, Abcam
E2F1	1:1000	3742, Cell Signaling
